# Immunotherapy in Pediatric Solid Tumors—A Systematic Review

**DOI:** 10.3390/cancers11122022

**Published:** 2019-12-14

**Authors:** Raoud Marayati, Colin H. Quinn, Elizabeth A. Beierle

**Affiliations:** Division of Pediatric Surgery, Department of Surgery, University of Alabama at Birmingham, Birmingham, AL 35294, USA; rmarayati@uabmc.edu (R.M.); chquinn@uab.edu (C.H.Q.)

**Keywords:** pediatric solid tumors, immunotherapy, chimeric antigen receptors, cancer vaccines, oncolytic viral therapy, immune checkpoint inhibitors, immunomodulation

## Abstract

Despite advances in the treatment of many pediatric solid tumors, children with aggressive and high-risk disease continue to have a dismal prognosis. For those presenting with metastatic or recurrent disease, multiple rounds of intensified chemotherapy and radiation are the typical course of action, but more often than not, this fails to control the progression of the disease. Thus, new therapeutics are desperately needed to improve the outcomes for these children. Recent advances in our understanding of both the immune system’s biology and its interaction with tumors have led to the development of novel immunotherapeutics as alternative treatment options for these aggressive malignancies. Immunotherapeutic approaches have shown promising results for pediatric solid tumors in early clinical trials, but challenges remain concerning safety and anti-tumor efficacy. In this review, we aim to discuss and summarize the main classes of immunotherapeutics used to treat pediatric solid tumors.

## 1. Introduction

Immunotherapy is being popularized as an approach to target pediatric cancer. This treatment modality has proven effective in pediatric hematological malignancies such as acute lymphocytic leukemia (ALL), but there remains much to be learned before we can harness the potential of immunotherapy in the treatment of solid tumors. Here, we examine two broad immunotherapy approaches that may be utilized for the treatment of pediatric solid tumors: direct utilization of the immune system properties and immune system modulation. Within each of these categories, we discuss the benefits and challenges of each therapy for solid tumors and specifically highlight the effects on pediatric populations. The overarching objective of this review is to discuss immunotherapies that are currently in use as well as those with potential future use in the treatment of pediatric solid tumors.

## 2. Direct Utilization of the Immune System

### 2.1. Oncolytic Virus-Based Therapy

Oncolytic virus-based therapy is an emerging approach designed to target a variety of cancers. The concept for utilizing oncolytic virotherapy in cancer treatment originated from observations that patients with Hodgkin’s lymphoma temporarily improved following a hepatitis infection [1]. Oncolytic viruses are constructed by altering the genetic profile of a viral vector to render the virus apathogenic while maintaining its ability to infect, replicate, and spread amongst host cells. Oncolytic viruses are also often engineered with specific receptors for cancer cells, rendering them target-specific and potentially more efficacious [2]. The cancer cells will then behave as hosts and will be subjected to the oncolytic effects of the virus.

The benefit of oncolytic viral therapy is twofold: (1) it harnesses a virus’s innate ability to lyse cancer cells and (2) it has the potential to trigger a cytotoxic immune response. In cancer cells, the upregulation of DNA replication assists in the production of viral progeny. The buildup of progeny results in lysis of the cells and infection of neighboring cancer cells [3]. This approach is effective for solid tumors, as viral delivery may be accomplished through direct intratumoral injections, resulting in direct killing of the malignant cells without producing severe systemic side effects or unwanted hepatic degradation of the virus, which may occur with systemic injection [4]. As a result of viral-mediated tumor cell lysis, pathogen-associated molecular patterns (PAMPs), damage-associated molecular patterns (DAMPs), and tumor-associated antigens (TAA) are released. These molecular signals initiate an immune response directed at the tumor even if this tumor has previously and successfully evaded the immune system [5]. These molecular signaling molecules allow for an intact immune system to utilize natural killer (NK) cells, dendritic cells (DCs), and other antigen-presenting cells (APCs) to directly target the cancer cells [6].

A variety of replicating viruses have been studied as cancer therapeutics, including adenoviruses, herpesviruses, paramyxoviruses, picornaviruses, poxviruses, reoviruses, rhabdoviruses, and togaviruses [7]. In pediatrics, variants of oncolytic Herpes simplex virus (oHSV) have been shown effective in a variety of solid tumors, such as glioblastoma, neuroblastoma, and sarcoma [8]. oHSVs have been genetically engineered to allow for selective uptake or replication of the virus by tumor cells but not healthy tissue [9,10]. Additionally, particular oHSVs have been engineered to produce chemokines or increased amounts of TAA, which stimulates and bolsters the immune system response directed toward the tumor [6,11].

There is great potential to use the immune response to target tumors through oHSV. NK cells are the first line of defense and will destroy the cancer cells or use cytokines to recruit other immune cells. Following this innate immune response, an adaptive response may ensue [12,13]. Such a reaction could potentially lead to immune memory, negating the need for retreatment and theoretically, tumor relapse. This built-in defense mechanism could then take over for the destruction of most of the tumor. Barriers to this response, especially in solid tumors, include complete viral clearance, dense fibrosis surrounding the tumor, and the tumor microenvironment (TME) [5]. Combination therapy provides a means to overcome these barriers. In melanoma, combining T-VEC, a modified herpes simplex virus, with a MEK inhibitor (trametinib) produced an increased infiltration of CD8+ T cells into the tumor and a decreased tumor size in vivo [14]. A pre-clinical investigation of the TME in sarcoma showed that modulation of tumor-associated macrophages (TAMs) could potentiate an immune response. This study focused on Ewing sarcoma and oHSV. The investigators demonstrated that by targeting the TME with trabectedin, a currently approved chemotherapeutic, the M2 macrophage population was decreased, allowing for uninhibited viral infection by the rRp450 virus. They also showed that combining rRp450 with trabectedin in an in vivo xenograft model of Ewing sarcoma significantly decreased tumor volume and increased animal survival [15]. Such studies provide an avenue for future investigations in viral therapy.

Currently, there are no commercially available viral therapies that are routinely used for extracranial solid tumors in pediatric patients. T-VEC is a viral therapy available for the treatment of melanoma in adults, but studies have not specifically tested its use in a pediatric-only population [16]. There are only four clinical trials listed on www.clinicaltrials.gov that are actively recruiting for viral therapy in pediatric cancers: oHSV in cerebellar tumors (NCT03911388), oHSV in supratentorial brain tumors (NCT02457845), adenovirus in gliomas (NCT03178032), and an oncolytic poliovirus in gliomas (NCT03043391). Considering there are 28 actively recruiting clinical trials listed for adults, there is obvious room for expansion of this therapeutic approach for pediatric solid tumors.

### 2.2. Antigen-Targeting Therapy

Tumor antigen-targeting therapy, initially based on antibody–drug conjugates (ADC), utilizes specific monoclonal antibodies (mAbs) as an approach to target cancer cells and cause antibody-dependent cell-mediated toxicity (ADCC). Initially, these mAbs were used to assist in more direct drug delivery of chemotherapeutics [17]. In pediatrics, this model has been employed to target a tumor-specific antigen, GD2, which is a di-ganglioside expressed on neuroblastoma and osteosarcoma. In these tumors, a specific antibody targeting GD2 has been developed and is used in clinical treatment [18,19]. Additionally, a newer target for neuroblastoma treatment is anaplastic lymphoma kinase (ALK). An mAb targeting the ALK surface receptor, mAb30/49, led to decreased tumor cell proliferation and viability in vitro [20].

In order to increase the efficacy of these antibodies, researchers are examining techniques to evoke the activity of immune cells, such as NK cells, to involve them in tumor cell lysis. Investigators have demonstrated this concept using an anti-GD2 antibody, hu14.18K322A, combined with IL-15. This combination resulted in decreased tumor cell viability in vitro and growth in vivo, as well as in an increase in mature NK cells in the TME [21].

To take advantage of the immune system, antibodies have been developed in combination with proteins designed to elicit an immune-stimulating response. FDA-approved drugs, such as dinutuximab (Ch14.18, murine/human chimeric antibody to GD2) and naxitamab (hu3F8, a humanized mAB to GD2), are administered in combination with granulocyte-macrophage colony stimulating factor (GM-CSF) to boost the immune response [22]. A phase I clinical trial studied naxitamab with GM-CSF and its effects on resistant neuroblastoma (NCT012757626). A total of 31 children had evaluable disease. Of those, 14 (45%) had a complete or partial tumor response, 5 (16%) had stable disease, and 11 (35%) had progressive disease [23]. To study the role of the immune system in more depth, researchers have utilized dinutuximab in a patient-derived xenograft (PDX) model. Investigators have shown that following surgical resection of neuroblastoma in a PDX model, increased animal survival and decreased tumor invasiveness were achieved with the administration of dinutuximab and activated NK cells [24]. For the mAb to achieve such an immune reaction independently, a different technology must be applied.

**Figure 1:**
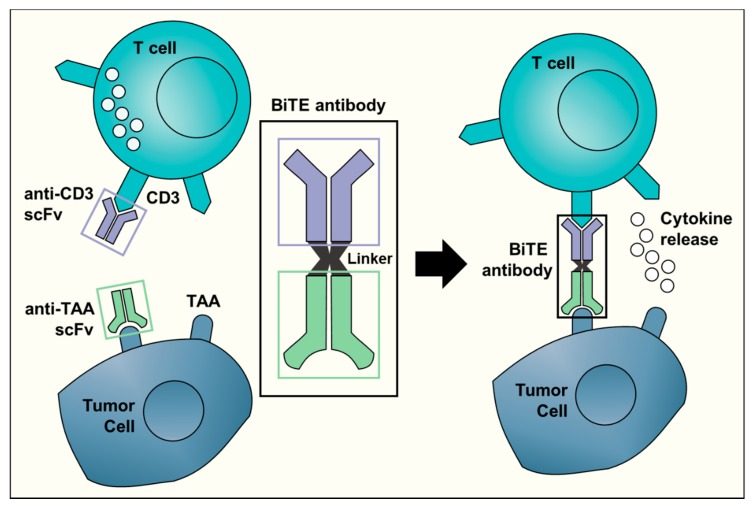
A schematic representation of the Bispecific T-cell Engager (BiTE) technology. The BiTE antibody connects the CD3 binding site on T cells with a tumor-associated antigen (TAA) specific to tumor cells. This triggers T cell activation and cytokine release, ultimately resulting in an anti-tumor response. The anti-CD3 single-chain variable fragment (scFv, shown in purple) is shared by all BiTE antibodies. The target antigen-specific scFv (in light green) is different for each BiTE antibody and can recognize targets such as CD19 or EpCAM.

Bispecific antibodies, unlike normal antibodies, elicit a cytotoxic T cell response against a specific tumor target. The Bispecific T Engager (BiTE) technology activates a T cell response by binding to CD3 on T cells [25]. The molecule combines the CD3 binding site with a second site that is tumor-specific. BiTE directly targets the cancer and limits damage to non-malignant tissue (Figure 1). The direct activation of cytotoxic T cells limits the need for other anti-cancer interventions. Currently, clinical trials with BiTE antibodies are limited to just two TAA: CD19 and EpCAM [26]. Of the two, only anti-CD19 (blinatumomab) has been investigated in children and it has been limited to hematologic malignancies. Blinatumomab was administered to children with relapsed/refractory ALL. In this phase I/II study, 39% of the children that received the determined dosage and treatment plan achieved a minimal residual disease response [27]. Elitzur and colleagues reported 11 pediatric patients with ALL who were treated with blinatumomab as a bridge to further therapy after suffering from severe chemotherapy toxicities. All 11 children went on to resume standard chemotherapy with an overall survival of 80% [28]. Further preclinical studies and clinical trials with other TAA for BiTE antibodies will be required before this promising technology may be translated for clinical use in the treatment of pediatric solid tumors.

### 2.3. Immune Checkpoint Inhibitors

Cytotoxic T-lymphocyte-associated antigen-4 (CTLA-4), the first immune-checkpoint receptor to be targeted clinically, is expressed on the surface of activated T cells and transmits an inhibitory signal to T cells. Normal T cell activation requires the engagement of the T cell receptor (TCR)/CD3 complex and the CD28 co-stimulatory signal, which then leads to increased expression of the co-inhibitory signal, CTLA-4. CTLA-4 binds to B7 molecules (CD80 and CD86) with greater affinity, thus out-competes CD28 for their shared ligands, preventing T cell activation. CTLA-4 signaling is utilized by some tumor cells to evade T cell anti-tumor activity. Thus, CTLA-4 blockade potentiates effective immune responses against tumor cells [29]. CTLA-4 is also found in regulatory T cells (Tregs) and contributes to their inhibitory function. CTLA-4 blockade in Tregs results in their decreased immunosuppression [30].

Preclinical data suggest that pediatric solid tumors have high expression of CTLA-4. In a panel of 34 adult and pediatric tumor cell lines, including osteosarcoma, rhabdomyosarcoma, and neuroblastoma, CTLA-4 expression was found at different densities on 88% of the cell lines examined, with higher intensity of staining in osteosarcoma [31]. In addition, 20 pediatric patients, 11 with osteosarcoma and 9 with Ewing sarcoma, had significantly increased expression of CTLA-4 on both CD4+ and CD8+ T cells obtained from peripheral blood samples compared to healthy controls [32]. These findings indicate that targeting CTLA-4 may be useful in these pediatric tumor types.

Ipilimumab is a mAb directed toward CTLA-4 signaling. Ipilimumab is FDA-approved for the treatment of adults and children with unresectable or metastatic melanoma. Recently, a phase I clinical trial (NCT01445379) included a total of 33 patients aged 2–21 years with recurrent or refractory solid tumors treated with CTLA-4 blockade. In this study, ipilimumab was well tolerated and resulted in increased activation of cytotoxic T lymphocyte without increased infiltration of Tregs; however, no objective tumor regression was observed [33].

Programmed cell death receptor 1 (PD-1) and its ligands (PD-L1 and PD-L2) are also part of the immune checkpoint pathway. PD-1 plays a role in downregulating T cell activation, which leads to tumor tolerance, while PD-Ls inhibit cytokine production and anti-tumor lymphocytes in the TME [34]. PD-1 is also highly expressed on Tregs and, when engaged by its ligand, is thought to enhance the activity and proliferation of these cells [35].

Several preclinical studies examined the expression of PD-1 and PD-L1 in pediatric cancer subtypes, with conflicting results. Only 9% of 451 pediatric tumors expressed PD-L1 in at least 1% of tumor cells, with the highest expressors being Burkitt lymphoma (80%), glioblastoma multiforme (36%), and neuroblastoma (14%) [36]. Conversely, in another study of children with advanced melanoma, relapsed or refractory solid tumors, or lymphoma, 33% of 689 screened tumors were positive for PD-L1 expression [37]. Of note, PD-L1 staining was associated with inferior survival among neuroblastoma patients [36], and higher expression of PD-1 correlated with disease progression in patients with osteosarcoma [38].

Pembrolizumab, an anti-PD-1 antibody, is FDA-approved for the treatment of both adults and children with refractory Hodgkin’s lymphoma. Nivolumab, another anti-PD-1 antibody, has shown responses in adult solid tumors [39,40]. In pediatric solid tumors, these therapies remain under investigation. In five children aged 3–7 years with brain tumors treated with pembrolizumab, all progressed, and the median survival was 3.2 months [41]. In a retrospective review of 10 children with recurrent or refractory brain tumors treated with nivolumab, 9 patients had radiographic disease progression. Three patients had partial response at the primary tumor site, of whom two had progression of metastatic disease [42]. In other small studies of nivolumab treatment in pediatric brain tumor patients, results were mixed. [43,44]. Currently, a phase I/II trial (NCT03585465) is assessing nivolumab in combination with chemotherapy in pediatric patients with refractory/relapsing solid tumors or lymphoma. Two other trials are evaluating nivolumab alone: NCT02992964 is a pilot study of nivolumab in pediatric patients with refractory/recurrent hypermutated malignancies, and NCT02901145 is evaluating nivolumab in progressive/relapsed pediatric solid tumors, including osteosarcoma, Ewing sarcoma, neuroblastoma, and rhabdomyosarcoma.

Dual checkpoint blockade is hypothesized to prevent immune escape and may be promising in the treatment of pediatric solid tumors. Combinations of CTLA-4 and PD-1 antibodies are currently being investigated [45]. In an implantable murine model of metastatic osteosarcoma, treatment with anti-PD-L1 antibody resulted in downregulation of PD-L1 expression and upregulation of CD80/CD86 expression on tumor cells, as well as upregulation of CTLA-4 on tumor infiltrating CD8+ T cells [46]. Furthermore, combination therapy of PD-1/CTLA-4 signaling blockade resulted in complete protection from metastasis in 50% of treated mice as well as in T cell memory protection against future tumor inoculation [46]. Currently, NCT02304458 is an ongoing phase I/II trial evaluating PD-1/CTLA-4 signaling blockade combination therapy in pediatric patients with relapsed/refractory solid tumors.

## 3. Modulation of the Immune System

### 3.1. Tumor Microenvironment: Cancer-Associated Fibroblasts, Tumor-Associated Macrophages, and Myeloid-Derived Suppressor Cells

Many non-tumor cells including macrophages and fibroblasts are present in the TME and affect the malignant potential of tumor cells. Cancer-associated fibroblasts (CAFs) and TAMs are two of the primary infiltrating stromal cells.

CAFs are activated fibroblasts that play an important role in promoting tumor growth, invasion, and angiogenesis [47]. In a study of 60 primary neuroblastoma tumors, increased CAFs were associated with significantly higher microvascular proliferation and Schwannian stroma-poor histopathology, both poor prognostic factors [48]. In addition, blocking CAF-derived prostaglandin E2 (PGE2) production with a small molecule inhibitor was shown to reduce neuroblastoma cell growth, impair angiogenesis, and reduce tumor growth in vivo [49]. Further, in a genetically modified murine lung carcinoma model, depletion of CAFs resulted in significant inhibition of tumor growth and enhanced anti-tumor immunity [50]. While CAFs could be a potential therapeutic target in pediatric solid tumors, there are currently no methods suitable for clinical translation, and further studies are needed to guide the development of such stroma-directed therapy.

TAMs, which most closely resemble M2 macrophages, are major contributors to the TME. Whether TAMs promote or impede tumor growth is tissue-dependent. High infiltration of TAMs was first described in neuroblastoma and shown to be associated with worse prognosis [51,52]. On the contrary, TAMs play a beneficial role in medulloblastoma and induced tumor growth suppression in vitro as well as in various mouse models [53]. Furthermore, the presence of TAMs, detected by genome-wide mRNA profiling and immunohistochemistry, was shown to be associated with suppression of metastasis and improved overall survival in patients with high-grade osteosarcoma [54], thus providing a rationale for the use of macrophage-activating agents such as liposomal muramyl tripeptide phosphatidylethanolamine (L-MTP-PE). L-MTP-PE is a synthetic analog of a bacterial wall component that induces the activation of monocytes and macrophages in the TME, thereby promoting their anti-tumor activity [55]. Conflicting results exist regarding the utility of L-MTP-PE [56]. A report from the Children’s Oncology Group (COG) analyzed whether the addition of L-MTP-PE would improve outcomes in patients with osteosarcoma and found a statistically significant improvement of the overall survival from 70 to 78% [57]. However, 91 patients with metastatic osteosarcoma were separately analyzed, with no significant survival difference seen with the administration of L-MTP-PE [58].

Cancer cells also recruit myeloid-derived suppressor cells (MDSCs) to the TME as a mechanism to successfully evade the immune system. MDSCs are a population of tumor-infiltrating cells with immune-suppressive and tumor-promoting activity. MDSCs suppress both adaptive and innate arms of immunity through direct inhibition of the cytotoxic functions of T cells and NK cells [59]. Inhibition of MDSCs in three different immunocompetent mouse models of neuroblastoma resulted in the inhibition of tumor growth [60]. Thus, immunotherapies aimed at eliminating this suppressor cell subset could be advantageous in targeting the TME by counteracting the tumor escape mechanism and resuscitating the immune system. There are currently no known such therapies for pediatric solid tumors.

### 3.2. Cytokines and Growth Factors

Cytokines and growth factors that influence immune cells’ proliferation, phenotype, or function remain under investigation with respect to treatment of pediatric solid tumors.

An example of anti-tumor cytokine therapy involves interleukin 2 (IL-2). IL-2 is a gamma-c cytokine produced by T helper 1 cells that functions to activate T cell proliferation and facilitate the maintenance of NK cells [61]. Currently, IL-2 is FDA-approved for treating adults with renal cell carcinoma (RCC) and malignant melanoma [62]. In children with large refractory sarcoma or neuroblastoma, several phase I and II trials utilizing IL-2 as monotherapy have shown no measurable anti-tumor effects, and relapses occurred despite immune activation [63,64,65]. Of note, one of five children with RCC had a complete response which was consistent with the 10–20% response rate observed in adults [64]. However, IL-2 administered with alternating cycles of GM-CSF plus the mAb ch14.18 (dinutuximab) resulted in higher rates of event-free (66% versus 46%) and overall (86% versus 75%) survival after 2 years compared to standard therapy alone in children with high-risk neuroblastoma [18].

Alpha-interferon (IFN-α) is another cytokine known to activate cytotoxic T lymphocytes and NK cells [66]. IFN-α is FDA-approved for use in adults to treat malignant melanoma, chronic myelogenous leukemia (CML), hairy cell leukemia, and acquired immunodeficiency syndrome (AIDS)-related Kaposi sarcoma. A limited number of studies have evaluated the use of IFN therapy to treat pediatric solid tumors. High-dose IFN-α administered for 4 weeks followed by a lower maintenance dose for 48 weeks was feasible in children with resected stage III melanoma and was associated with less toxicity than in adults treated with the same regimen [67]. However, 2 out of 15 patients were taken off that study for recurrent disease during maintenance therapy [67]. A phase II study (NCT00041145) of pegylated IFN-α in 32 children with diffuse intrinsic pontine glioma (DIPG) reported prolonged median time to progression without significant improvement of the two-year survival rate [68], concluding that monotherapy with pegylated IFN-α may not be adequate, and further evaluation for use in combination studies is needed. Recently, a phase II trial (NCT00678951) explored the effect of IFN-α in children with unresectable plexiform neurofibromas and found both clinical and radiographic improvements; weekly injections of IFN-α resulted in at least doubling of the time to progression [69]. Currently, a combination of pegylated IFN-α, chemotherapy, and surgery is being tested in a phase III COG trial to treat patients, including children over the age of 5, with high-grade osteosarcoma (NCT00134030).

The cytokine receptor activator of nuclear factor-κB ligand (RANKL) is a member of the tumor necrosis factor (TNF) family that, in addition to being expressed on the surface of osteoblasts, is released by activated T lymphocytes [70]. RANKL regulates bone metabolism and plays a role in the pathophysiology of bone metastasis. RANKL induces osteoclast activation, which then mediates bone resorption and release of growth factors, resulting in a cycle of bone destruction and tumor proliferation [71]. Denosumab, a RANKL antibody, inhibits this osteoclast-mediated bone destruction. It has been used in phase II clinical trials in adults with multiple myeloma and metastatic breast and prostate cancer where it suppressed bone resorption [72]. In a study of 40 patients including 14 children, RANKL was expressed in 75% of high-grade osteosarcomas, and its expression correlated with a more aggressive clinical course, poor response to neoadjuvant chemotherapy, and poor event-free survival [73]. Denosumab may thus have a utility in the treatment of osteosarcoma and is currently being evaluated in a phase II clinical trial (NCT02470091) in children with recurrent or refractory osteosarcoma.

TNF, a peptide produced by macrophages and lymphocytes, has cytostatic and cytolytic effects on tumor cells in vitro [74] as well as stimulates necrosis and tumor regression in vivo [75]. Therapies incorporating recombinant TNF have been limited by the development of systemic toxicities, including hypotension, hemorrhagic gastritis, hyperbilirubinemia, and elevated creatinine [76]. Recombinant TNF has been studied in combination with dactinomycin in a phase I trial in 21 patients with refractory malignancies, including sarcoma and Wilms tumor. Evidence of anti-tumor activity was observed in only three patients, including one with Wilms tumor. Based on the anti-tumor activity observed in that patient with Wilms tumor, a phase II trial evaluated the combination of TNF and dactinomycin in patients with relapsed or refractory Wilms tumor. The combination was well tolerated and resulted in complete response in 16% and stable disease in 26% of patients [77].

Tumor necrosis factor-related apoptosis-inducing ligand (TRAIL) is another member of the TNF superfamily. TRAIL activates death receptors expressed on tumor cells, such as TRAIL-R1 and TRAIL-R2, inducing apoptosis [78]. Osteosarcoma, Ewing sarcoma, and rhabdomyosarcoma cell lines that express TRAIL death receptor were found to be sensitive to TRAIL-mediated apoptosis [79,80]. Lexatumumab, an agonistic human mAB against TRAIL-R2, binds and activates TRAIL-R2. Lexatumumab was evaluated in a phase I trial (NCT00428272) in pediatric patients with recurrent or progressive solid tumors, including osteosarcoma, Ewing sarcoma, rhabdomyosarcoma, soft tissue sarcoma, hepatoblastoma, and nephroblastoma [81]. While no patients experienced either a complete or a partial response, several showed evidence of anti-tumor activity. A patient with osteosarcoma demonstrated resolution of the clinical symptoms and positron emission tomography activity, ongoing for more than 1 year off therapy, while a patient with hepatoblastoma showed a dramatic biomarker response [81].

GM-CSF is a myeloid growth factor that stimulates hematopoietic stem cells to make granulocytes and monocytes. In acute myeloid leukemia (AML), GM-CSF led to sensitization of leukemic cells and enhanced the cytotoxicity effects of chemotherapy [82]. Inhaled GM-CSF was evaluated in three adolescents with pulmonary metastases from osteosarcoma and Ewing sarcoma (NCT00673179). There were virtually no toxicities, and a patient with Ewing sarcoma demonstrated a complete response [83].

Other cytokines studied include IFN-γ, which is produced by NK cells and T cells in response to viral and intracellular bacterial infections as well as during anti-tumor responses. IFN-γ is currently FDA-approved for the treatment of children with osteopetrosis and chronic granulomatous disease. In addition, INF-γ has shown activity against Ewing sarcoma in combination with a TRAIL agonist in preclinical models [84] and has potential for clinical translation.

### 3.3. Chimeric Antigen Receptor T Cell Therapy

Chimeric antigen receptor (CAR) T cell therapy has been rapidly expanding in pediatric cancer therapeutics. In this approach, autologous T cells are collected from the patient, expanded, and subsequently engineered to express CARs, which are designed to redirect T cells to a selected tumor antigen. This non-physiologic T cell activation bypasses the need for tumor antigen presentation to major histocompatibility complex (MHC) Class I molecules, which are often downregulated in cancer, and allows antigen-expressing malignant cells to be recognized and destroyed by the CAR-redirected T cells. Different generations of CAR T cells exist. The first generations carry a single-chain fragment variable region (scFv) or activation domain against a TAA [85], while the second and third generations involve the addition of one or two co-stimulatory molecules, such as CD28, CD137 (4-1 BB), and/or CD134 (OX-40), and show improved T cell proliferation and survival and anti-tumor effects [86,87,88].

The majority of studies involving CAR T cells in the pediatric population were aimed at hematological malignancies, with fewer designed for malignant solid tumors [89]. Neuroblastoma was the first pediatric solid tumor on which CAR T cells have been tested in clinical trials [90,91]. In a phase I trial using first-generation CAR T cells targeting GD2 in refractory neuroblastoma, there was a 45% response rate in patients with active disease. Three of 11 (27%) patients achieved complete remission, with 2 achieving sustained remission for more than 5 years [90]. Currently, third-generation anti-GD2 CAR T cells which integrate the CD28 and OX-40 costimulatory domains are undergoing a phase I study (NCT01822652) for patients with refractory neuroblastoma [92]. The development of CAR T cells targeting ALK has also been suggested. Although no clinical trials have yet been initiated, human anti-ALK CAR T cells were shown to eradicate ALK-positive neuroblastoma tumors in a xenogeneic immunodeficient murine model [93]. However, the efficacy of these CAR T cells was dependent on both target tumor antigen and CAR receptor density [93].

HER2/Neu, which is highly expressed in medulloblastoma, osteosarcoma, and nephroblastoma, has also been incorporated into CAR T cells [94,95]. HER2-specific CAR T cells efficiently recognize and eliminate tumor cells even with modest levels of HER2 expression [96,97] and have been tested in a preclinical model of osteosarcoma [98]. In a phase I clinical trial utilizing anti-HER2 CAR T cells in 19 patients with advanced pediatric sarcoma (NCT00902044), 4 had stable disease for 3 to 14 months, and the median overall survival was 10.3 months [99].

Other CAR T cells being evaluated in clinical trials include those targeting interleukin 13 receptor alpha (IL-13Rα), which is shown to be overexpressed in gliomas and other pediatric brain tumors [100,101]. IL13Rα2-specific CAR T cells targeted and killed high-grade glioma cells and glioma stem-like initiating cells in vitro [102], as well as caused the regression of established human glioblastoma orthotopic xenografts [103]. Currently, no trials utilizing IL13Rα2-specific CAR T cells are actively enrolling pediatric patients.

While dramatic clinical responses were seen in clinical trials, significant potential toxicities were associated with the use of CAR T cell therapy. Cytokine release syndrome (CRS) has been the most commonly described severe toxicity and is characterized by fever, tachycardia, hypotension, and hypoxia. Reports of CRS ranged from mild flu-like symptoms to life-threatening multi-organ system failure [104]. This constellation of inflammatory symptoms results from the release of cytokines from the CAR T cells and other immune cells. A variety of neurotoxicities have also been reported with CAR T cell therapy, ranging from somnolence, tremors, and seizures to cerebral edema and death [104]. Models utilizing serum cytokine levels after CAR T cell infusion are being developed to predict those at risk for severe CRS or neurotoxicity and may guide future interventions with immunosuppression or cytokine-directed therapy [105]. Approaches to ameliorate CRS or neurotoxicity while maintaining treatment efficacy include directly targeting specific cytokines, such as IL6 blockade by tocilizumab [106], as well as the use of an inducible caspase suicide safety switch that may be activated, leading to programmed cell death to prevent unanticipated toxicities [107]. The latter has been tested in a clinical trial for pediatric patients and led to the elimination of 90% of CAR T cells within 30 minutes of the infusion [108]. Another type of toxicity associated with CAR T cell therapy is agammaglobulinemia, which may be corrected with gammaglobulin replacement.

### 3.4. Natural Killer Cell-Based Immunotherapy

NK cells have been investigated as potential immunotherapeutics due to their anti-tumor effects, through either direct cytotoxicity or antibody-dependent cellular toxicity. A major component of NK cell target recognition depends on the surveillance of human leukocyte antigen (HLA) class I molecules by killer immunoglobulin-like receptors (KIRs) [109]. KIRs are expressed on the surface of NK cells and transmit immune inhibitory signals to maintain tolerance to NK cells. Cancer cells without an inhibitory HLA ligand may trigger NK cell activation.

The potential for the therapeutic application of NK cells was primarily tested in hematologic malignancies, such as AML and ALL [110]. In these studies, reduced risk of relapse and improved survival were observed after allogeneic hematopoietic stem cell transplantation (HSCT) when HLA ligands against the inhibitory KIRs present in the donor were absent in the recipient. HSCT has also been proposed as a potential curative alternative in children with refractory solid tumors, such as Ewing sarcoma [111], neuroblastoma [112], melanoma [113], and hepatoblastoma [114]. Perez-Martinez et al. suggested that the clinically beneficial graft-versus-tumor (GVT) effect seen after HSCT may be mediated by donor–recipient inhibitory KIR–HLA mismatched NK cells [115]. That study examined three children with refractory solid tumors and observed a clinical response in the two patients with a KIR–HLA mismatched donor during the time when NK cells were the major lymphocyte population. In addition, the degree of tumor response appeared to correlate with the number of KIR-activating receptors [115].

A few ongoing early clinical trials are investigating the role of autologous and allogeneic NK cells in pediatric solid tumors. NCT01875601 is employing ex vivo activated and expanded autologous NK cells with recombinant human IL-15 in children with brain tumors, sarcoma, Wilms tumor, and rhabdomyosarcoma after lympho-depleting chemotherapy. Two trials (NCT01576692 and NCT01857934) are exploring the safety and feasibility of allogenic NK cell infusions from haploidentical donors in children with high-risk neuroblastoma in combination with the humanized anti-GD2 antibody (hu14.18K322A) and standard chemotherapy. NCT01287104 is assessing the feasibility and toxicity of infusing escalating doses of donor-derived activated NK cell donor lymphocyte infusions (NK-DLI) following HLA-matched T cell-depleted (TCD) peripheral blood stem cell transplant (PBSCT) in patients with metastatic or recurrent pediatric solid tumors. NCT00640796 was recently completed. This phase I study was designed to determine the safety of infusing expanded NK cells, obtained from a patient’s family member with partial HLA mismatch, into pediatric patients with Ewing sarcoma family of tumors (ESFT) and rhabdomyosarcoma. The results of this trial are not yet available.

The efficacy of NK cell-based immunotherapy may be reduced by numerous factors such as limited in vivo proliferation and the immunosuppressive milieu of the TME. Furthermore, tumor cells develop various strategies to evade NK cell attack or to impair the activity and function of NK cell therapy. For example, tumor cells often upregulate the expression of KIR ligands, such as HLA-G [116]. HLA-G may inhibit the proliferation and cytotoxicity of NK cells [117]. Ectopic HLA-G expression on Ewing sarcoma suppressed the activity of GD2-specific CAR-expressing NK cells [118]. In addition, blocking of HLA-G on tumor cells in patients with chronic lymphocytic leukemia (CLL) increased their susceptibility to NK cell-mediated cytotoxicity [119]. Strategies to augment the anti-tumor efficacy of NK cells, prolong their survival and persistence in vivo, and restore their functions from exhaustion in the TME will maximize the effects of this novel therapy. Furthermore, NK cells have not been associated with significant off-target effects, graft-versus-host disease, or CRS [120], making this therapy an attractive modality to explore.

### 3.5. Cancer Vaccines

Vaccines are some of the oldest means for modulating the immune response. The idea behind this therapy is that an exposure to a pathogen will allow for the generation of an adaptive immune response toward future re-exposure to that pathogen. For cancer, this same idea has also been explored in hopes of generating an anti-tumor response and cell-mediated immunity [5]. In comparison to other immunotherapies, this manipulation of the immune system shows promise specifically for pediatric solid tumors but has had limited study.

Anti-cancer vaccines typically exploit DCs. These antigen-presenting cells serve an important role as a bridge between the adaptive and the innate immune response, allowing for both an active and a passive attack on the tumor [121,122]. Different mechanisms used to stimulate T cell responses from DCs include mRNA strands, cell surface receptors, and lysed intracellular proteins. Researchers proved this point by pulsing DCs with sarcoma cell lysate and priming with cytokines. These DCs were administered to mice for immunization. They found these cells adept at producing a primary T cell response as well as significantly decreased pulmonary metastasis of the sarcoma tumor cells [123]. In pediatrics, a rhabdomyosarcoma cell line known as M3-9-M was grown in vivo and tested similarly with a vaccine. The authors were able to demonstrate a decrease in tumor growth in vivo, and upon depletion of CD4+ and CD8+ T cells, a lack of tumor cell response, indicating the necessity of a T cell response for anti-tumor effects [124].

In clinical trials, vaccines have been an area of focus for the treatment of gliomas, neuroblastoma, sarcoma, and Wilms tumor. In one clinical trial, dendritic vaccines were administered to pediatric patients with solid tumors, with one patient achieving a significant decrease in tumor size, and two patients showing undetectable disease. The tumor lysates were tested for immune response. Compared to pre-vaccine samples, the post-vaccine tumor lysates had a significantly higher level of IFN-γ, thus indicating the effectiveness of this vaccine at producing an immune response [125]. This study also demonstrated that dendritic cell-based vaccines could be administered in the outpatient setting and were not associated with significant toxicities in children [125]. Many of the future directions of the use of dendritic cell-based vaccines are toward improving efficacy through further immunomodulation [121]. Other clinical trials are studying the combination of vaccines with chemotherapeutics. A phase I/II trial for relapsed or refractory neuroblastoma and sarcoma used decitabine and DC/MAGE-A1, MAGE-A3, and NY-ESO-1 peptide vaccines (NCT01241162). Using CD137 as a T cell marker, 6 out of the 10 patients given the vaccine had a T cell response. Of those six patients, one had a complete tumor response, while one remains disease-free two years after the trial [126,127]. Such trials are the groundbreaking work that is needed to further implement these therapies and determine what is most effective in the pediatric population.

## 4. Conclusions

Immunotherapeutics with either direct utilization or modulation of the immune system provide novel treatment approaches for the treatment of children with solid tumors (Table 1). Although these therapies have shown promising clinical results, they are currently utilized in a limited number of pediatric cancer diagnoses. A more generalized pediatric use will require further studies to firmly establish the safety and treatment efficacy of these approaches and identify ways to integrate them with current conventional treatment regimens for a greater impact in pediatric solid tumors.

## Figures and Tables

**Table 1:** Immunotherapy clinical trials for pediatric solid tumors discussed in this review.

Immunotherapy Approach	Disease	Target	Agent/Compound	NCT #	Phase of Study
Viral therapy	Cerebellar Brain Tumor	N/A	G207 (HSV)	03911388	Phase I (recruiting)
Viral therapy	Supratentorial Brain Tumor	N/A	G207 (HSV) +/− radiation	02457845	Phase I (recruiting)
Viral therapy	DIPG	N/A	DNX-2401 (adenovirus)	03178032	Phase I (recruiting)
Viral therapy	Glioma	N/A	Recombinant Polio/Rhinovirus	03043391	Phase I (recruiting)
Antigen-targeting and growth factor therapy	Neuroblastoma	GD2	hu3F8 (mAB against GD2) and GM-CSF	01757626	Phase I/II (recruiting)
Immune checkpoint inhibitor	Solid tumors	CTLA-4	Ipilimumab	01445379	Phase I (completed)
Immune checkpoint inhibitor	Solid tumors or lymphoma	PD-1	Nivolumab with chemotherapy	03585465	Phase I/II (recruiting)
Immune checkpoint inhibitor	Hypermutated malignancies	PD-1	Nivolumab	02992964	Phase I/II (recruiting)
Immune checkpoint inhibitor	Solid tumors	PD-1	Nivolumab	02901145	Phase I/II (not yet recruiting)
Immune checkpoint inhibitor	Solid tumors or sarcoma	PD-1/CTLA-4	Nivolumab +/− ipilimumab	02304458	Phase I/II (recruiting)
Cytokine therapy	DIPG	N/A	Pegylated IFN-α2b	00041145	Phase II (completed)
Cytokine therapy	Plexiform neurofibroma	N/A	Pegylated IFN-α2b	00678951	Phase II (completed)
Cytokine therapy	Osteosarcoma	N/A	Pegylated IFN-α2b	00134030	Phase III (active, not recruiting)
Cytokine targeted therapy	Osteosarcoma	RANKL	Denosumab (mAB against RANKL)	02470091	Phase II (active, not recruiting)
Cytokine targeted therapy	Solid tumors	TRAIL-R2	Lexatumumab (mAB against TRAIL-R2)	00428272	Phase I (terminated)
Growth factor therapy	Osteosarcoma, Ewing sarcoma	N/A	Inhaled GM-CSF (Sargramostim)	00673179	Phase I (terminated)
CAR T cells	Neuroblastoma	GD2	Anti-GD2 CAR T cells	01822652	Phase I (active, not recruiting)
CAR T cells	Sarcoma	HER2	Anti-HER2 CAR T cells	00902044	Phase I (recruiting)
NK cells with cytokine therapy	Brain tumors, sarcoma, Wilms tumor, RMS	N/A	NK cells +/− rhIL-15 after lympho-depletion	01875601	Phase I (completed)
NK cells with antigen targeted therapy	Neuroblastoma	GD2	hu14.18K322A (anti-GD2), NK cells	01576692	Phase I (completed)
NK cells with antigen targeted therapy	Neuroblastoma	GD2	hu14.18K322A (anti-GD2), NK cells	01857934	Phase II (active, not recruiting)
NK cells	Solid tumors	N/A	NK cells	01287104	Phase I (completed)
NK cells	Ewing sarcoma, RMS	N/A	NK cells	00640796	Phase I (completed)
Cancer Vaccine	Neuroblastoma, sarcoma, RMS	Cancer testes antigen	Decitabine and DC vaccine + adjuvant	01241162	Phase I (Completed)

#, number; HSV, Herpes simplex Virus; DIPG, diffuse intrinsic pontine glioma; NK, natural killer; hu3F8, humanized 3F8; mAB, monoclonal antibody; GM-CSF, granulocyte-macrophage colony stimulating factor; CTLA-4, cytotoxic T-lymphocyte-associated antigen-4; PD-1, programmed cell death receptor 1; IFN, interferon; RANKL, receptor activator of nuclear factor-κB ligand; TRAIL-R, tumor necrosis factor-related apoptosis-inducing ligand receptor; CAR, chimeric antigen receptor; RMS, rhabdomyosarcoma; rhIL-15, recombinant human interleukin 15; DC, dendritic cell.

## References

[1] Kelly E., Russell S.J. (2007). History of oncolytic viruses: Genesis to genetic engineering. Mol. Ther..

[2] Sze D.Y., Reid T.R., Rose S.C. (2013). Oncolytic virotherapy. J. Vasc. Interv. Radiol..

[3] Coffey M.C., Strong J.E., Forsyth P.A., Lee P.W. (1998). Reovirus therapy of tumors with activated Ras pathway. Science.

[4] Sanchala D.S., Bhatt L.K., Prabhavalkar K.S. (2017). Oncolytic Herpes Simplex Viral Therapy: A Stride toward Selective Targeting of Cancer Cells. Front. Pharmacol..

[5] Wedekind M.F., Denton N.L., Chen C.Y., Cripe T.P. (2018). Pediatric Cancer Immunotherapy: Opportunities and Challenges. Paediatr Drugs.

[6] Yin J., Markert J.M., Leavenworth J.W. (2017). Modulation of the Intratumoral Immune Landscape by Oncolytic Herpes Simplex Virus Virotherapy. Front. Oncol..

[7] Pol J.G., Levesque S., Workenhe S.T., Gujar S., Le Boeuf F., Clements D.R., Fahrner J.E., Fend L., Bell J.C., Mossman K.L. (2018). Trial Watch: Oncolytic viro-immunotherapy of hematologic and solid tumors. Oncoimmunology.

[8] Waters A.M., Friedman G.K., Ring E.K., Beierle E.A. (2016). Oncolytic virotherapy for pediatric malignancies: Future prospects. Oncolytic Virother..

[9] Tong Y., Qian W. (2014). Targeting cancer stem cells with oncolytic virus. Stem. Cell Investig..

[10] Foreman P.M., Friedman G.K., Cassady K.A., Markert J.M. (2017). Oncolytic Virotherapy for the Treatment of Malignant Glioma. Neurotherapeutics.

[11] Bridle B.W., Stephenson K.B., Boudreau J.E., Koshy S., Kazdhan N., Pullenayegum E., Brunelliere J., Bramson J.L., Lichty B.D., Wan Y. (2010). Potentiating cancer immunotherapy using an oncolytic virus. Mol. Ther..

[12] Cantoni C., Grauwet K., Pietra G., Parodi M., Mingari M.C., Maria A.D., Favoreel H., Vitale M. (2015). Role of NK cells in immunotherapy and virotherapy of solid tumors. Immunotherapy.

[13] Li Y., Yin J., Li T., Huang S., Yan H., Leavenworth J., Wang X. (2015). NK cell-based cancer immunotherapy: From basic biology to clinical application. Sci. China Life Sci..

[14] Bommareddy P.K., Aspromonte S., Zloza A., Rabkin S.D., Kaufman H.L. (2018). MEK inhibition enhances oncolytic virus immunotherapy through increased tumor cell killing and T cell activation. Sci. Transl. Med..

[15] Denton N.L., Chen C.Y., Hutzen B., Currier M.A., Scott T., Nartker B., Leddon J.L., Wang P.Y., Srinivas R., Cassady K.A. (2018). Myelolytic Treatments Enhance Oncolytic Herpes Virotherapy in Models of Ewing Sarcoma by Modulating the Immune Microenvironment. Mol. Ther. Oncolytics.

[16] Rehman H., Silk A.W., Kane M.P., Kaufman H.L. (2016). Into the clinic: Talimogene laherparepvec (T-VEC), a first-in-class intratumoral oncolytic viral therapy. J. Immunother. Cancer.

[17] Vedi A., Ziegler D.S. (2014). Antibody therapy for pediatric leukemia. Front. Oncol..

[18] Yu A.L., Gilman A.L., Ozkaynak M.F., London W.B., Kreissman S.G., Chen H.X., Smith M., Anderson B., Villablanca J.G., Matthay K.K. (2010). Anti-GD2 antibody with GM-CSF, interleukin-2, and isotretinoin for neuroblastoma. N. Engl. J. Med..

[19] Heiner J.P., Miraldi F., Kallick S., Makley J., Neely J., Smith-Mensah W.H., Cheung N.K. (1987). Localization of GD2-specific monoclonal antibody 3F8 in human osteosarcoma. Cancer Res..

[20] Carpenter E.L., Haglund E.A., Mace E.M., Deng D., Martinez D., Wood A.C., Chow A.K., Weiser D.A., Belcastro L.T., Winter C. (2012). Antibody targeting of anaplastic lymphoma kinase induces cytotoxicity of human neuroblastoma. Oncogene.

[21] Nguyen R., Moustaki A., Norrie J.L., Brown S., Akers W.J., Shirinifard A., Dyer M.A. (2019). Interleukin-15 Enhances Anti-GD2 Antibody-Mediated Cytotoxicity in an Orthotopic PDX Model of Neuroblastoma. Clin. Cancer Res..

[22] Ploessl C., Pan A., Maples K.T., Lowe D.K. (2016). Dinutuximab: An Anti-GD2 Monoclonal Antibody for High-Risk Neuroblastoma. Ann. Pharmacother..

[23] Kushner B.H., Cheung I.Y., Modak S., Basu E.M., Roberts S.S., Cheung N.K. (2018). Humanized 3F8 Anti-GD2 Monoclonal Antibody Dosing With Granulocyte-Macrophage Colony-Stimulating Factor in Patients With Resistant Neuroblastoma: A Phase 1 Clinical Trial. JAMA Oncol..

[24] Barry W.E., Jackson J.R., Asuelime G.E., Wu H.W., Sun J., Wan Z., Malvar J., Sheard M.A., Wang L., Seeger R.C. (2019). Activated Natural Killer Cells in Combination with Anti-GD2 Antibody Dinutuximab Improve Survival of Mice after Surgical Resection of Primary Neuroblastoma. Clin. Cancer Res..

[25] Baeuerle P.A., Reinhardt C. (2009). Bispecific T-cell engaging antibodies for cancer therapy. Cancer Res..

[26] Hoffman L.M., Gore L. (2014). Blinatumomab, a Bi-Specific Anti-CD19/CD3 BiTE((R)) Antibody for the Treatment of Acute Lymphoblastic Leukemia: Perspectives and Current Pediatric Applications. Front. Oncol..

[27] von Stackelberg A., Locatelli F., Zugmaier G., Handgretinger R., Trippett T.M., Rizzari C., Bader P., O’Brien M.M., Brethon B., Bhojwani D. (2016). Phase I/Phase II Study of Blinatumomab in Pediatric Patients With Relapsed/Refractory Acute Lymphoblastic Leukemia. J. Clin. Oncol..

[28] Elitzur S., Arad-Cohen N., Barzilai-Birenboim S., Ben-Harush M., Bielorai B., Elhasid R., Feuerstein T., Gilad G., Gural A., Kharit M. (2019). Blinatumomab as a bridge to further therapy in cases of overwhelming toxicity in pediatric B-cell precursor acute lymphoblastic leukemia: Report from the Israeli Study Group of Childhood Leukemia. Pediatr. Blood Cancer.

[29] Leach D.R., Krummel M.F., Allison J.P. (1996). Enhancement of antitumor immunity by CTLA-4 blockade. Science.

[30] Walker L.S. (2013). Treg and CTLA-4: Two intertwining pathways to immune tolerance. J. Autoimmun..

[31] Contardi E., Palmisano G.L., Tazzari P.L., Martelli A.M., Fala F., Fabbi M., Kato T., Lucarelli E., Donati D., Polito L. (2005). CTLA-4 is constitutively expressed on tumor cells and can trigger apoptosis upon ligand interaction. Int. J. Cancer.

[32] Hingorani P., Maas M.L., Gustafson M.P., Dickman P., Adams R.H., Watanabe M., Eshun F., Williams J., Seidel M.J., Dietz A.B. (2015). Increased CTLA-4(+) T cells and an increased ratio of monocytes with loss of class II (CD14(+) HLA-DR(lo/neg)) found in aggressive pediatric sarcoma patients. J. Immunother. Cancer.

[33] Merchant M.S., Wright M., Baird K., Wexler L.H., Rodriguez-Galindo C., Bernstein D., Delbrook C., Lodish M., Bishop R., Wolchok J.D. (2016). Phase I Clinical Trial of Ipilimumab in Pediatric Patients with Advanced Solid Tumors. Clin. Cancer Res..

[34] Brahmer J.R., Tykodi S.S., Chow L.Q., Hwu W.J., Topalian S.L., Hwu P., Drake C.G., Camacho L.H., Kauh J., Odunsi K. (2012). Safety and activity of anti-PD-L1 antibody in patients with advanced cancer. N. Engl. J. Med..

[35] Pardoll D.M. (2012). The blockade of immune checkpoints in cancer immunotherapy. Nat. Rev. Cancer.

[36] Majzner R.G., Simon J.S., Grosso J.F., Martinez D., Pawel B.R., Santi M., Merchant M.S., Geoerger B., Hezam I., Marty V. (2017). Assessment of programmed death-ligand 1 expression and tumor-associated immune cells in pediatric cancer tissues. Cancer.

[37] Geoerger B., Kang H.J., Yalon-Oren M., Marshall L.V., Vezina C., Pappo A.S., Laetsch T.W., Petrilli A.S., Ebinger M., Toporski J. (2018). KEYNOTE-051: An update on the phase 2 results of pembrolizumab (pembro) in pediatric patients (pts) with advanced melanoma or a PD-L1–positive advanced, relapsed or refractory solid tumor or lymphoma. J. Clin. Oncol..

[38] Zheng W., Xiao H., Liu H., Zhou Y. (2015). Expression of programmed death 1 is correlated with progression of osteosarcoma. APMIS.

[39] Robert C., Schachter J., Long G.V., Arance A., Grob J.J., Mortier L., Daud A., Carlino M.S., McNeil C., Lotem M. (2015). Pembrolizumab versus Ipilimumab in Advanced Melanoma. N. Engl. J. Med..

[40] Topalian S.L., Hodi F.S., Brahmer J.R., Gettinger S.N., Smith D.C., McDermott D.F., Powderly J.D., Carvajal R.D., Sosman J.A., Atkins M.B. (2012). Safety, activity, and immune correlates of anti-PD-1 antibody in cancer. N. Engl. J. Med..

[41] Blumenthal D.T., Yalon M., Vainer G.W., Lossos A., Yust S., Tzach L., Cagnano E., Limon D., Bokstein F. (2016). Pembrolizumab: First experience with recurrent primary central nervous system (CNS) tumors. J. Neurooncol..

[42] Gorsi H.S., Malicki D.M., Barsan V., Tumblin M., Yeh-Nayre L., Milburn M., Elster J.D., Crawford J.R. (2019). Nivolumab in the Treatment of Recurrent or Refractory Pediatric Brain Tumors: A Single Institutional Experience. J. Pediatr. Hematol. Oncol..

[43] Bouffet E., Larouche V., Campbell B.B., Merico D., de Borja R., Aronson M., Durno C., Krueger J., Cabric V., Ramaswamy V. (2016). Immune Checkpoint Inhibition for Hypermutant Glioblastoma Multiforme Resulting From Germline Biallelic Mismatch Repair Deficiency. J. Clin. Oncol..

[44] Zhu X., McDowell M.M., Newman W.C., Mason G.E., Greene S., Tamber M.S. (2017). Severe cerebral edema following nivolumab treatment for pediatric glioblastoma: Case report. J. Neurosurg. Pediatr..

[45] Twyman-Saint Victor C., Rech A.J., Maity A., Rengan R., Pauken K.E., Stelekati E., Benci J.L., Xu B., Dada H., Odorizzi P.M. (2015). Radiation and dual checkpoint blockade activate non-redundant immune mechanisms in cancer. Nature.

[46] Lussier D.M., Johnson J.L., Hingorani P., Blattman J.N. (2015). Combination immunotherapy with alpha-CTLA-4 and alpha-PD-L1 antibody blockade prevents immune escape and leads to complete control of metastatic osteosarcoma. J. Immunother. Cancer.

[47] Sounni N.E., Noel A. (2013). Targeting the tumor microenvironment for cancer therapy. Clin. Chem..

[48] Zeine R., Salwen H.R., Peddinti R., Tian Y., Guerrero L., Yang Q., Chlenski A., Cohn S.L. (2009). Presence of cancer-associated fibroblasts inversely correlates with Schwannian stroma in neuroblastoma tumors. Mod. Pathol..

[49] Kock A., Larsson K., Bergqvist F., Eissler N., Elfman L.H.M., Raouf J., Korotkova M., Johnsen J.I., Jakobsson P.J., Kogner P. (2018). Inhibition of Microsomal Prostaglandin E Synthase-1 in Cancer-Associated Fibroblasts Suppresses Neuroblastoma Tumor Growth. EBioMedicine.

[50] Kraman M., Bambrough P.J., Arnold J.N., Roberts E.W., Magiera L., Jones J.O., Gopinathan A., Tuveson D.A., Fearon D.T. (2010). Suppression of antitumor immunity by stromal cells expressing fibroblast activation protein-alpha. Science.

[51] Asgharzadeh S., Salo J.A., Ji L., Oberthuer A., Fischer M., Berthold F., Hadjidaniel M., Liu C.W., Metelitsa L.S., Pique-Regi R. (2012). Clinical significance of tumor-associated inflammatory cells in metastatic neuroblastoma. J. Clin. Oncol..

[52] Song L., Asgharzadeh S., Salo J., Engell K., Wu H.W., Sposto R., Ara T., Silverman A.M., DeClerck Y.A., Seeger R.C. (2009). Valpha24-invariant NKT cells mediate antitumor activity via killing of tumor-associated macrophages. J. Clin. Investig..

[53] Maximov V., Chen Z., Wei Y., Robinson M.H., Herting C.J., Shanmugam N.S., Rudneva V.A., Goldsmith K.C., MacDonald T.J., Northcott P.A. (2019). Tumour-associated macrophages exhibit anti-tumoural properties in Sonic Hedgehog medulloblastoma. Nat. Commun..

[54] Buddingh E.P., Kuijjer M.L., Duim R.A., Burger H., Agelopoulos K., Myklebost O., Serra M., Mertens F., Hogendoorn P.C., Lankester A.C. (2011). Tumor-infiltrating macrophages are associated with metastasis suppression in high-grade osteosarcoma: A rationale for treatment with macrophage activating agents. Clin. Cancer Res..

[55] Mori K., Ando K., Heymann D. (2008). Liposomal muramyl tripeptide phosphatidyl ethanolamine: A safe and effective agent against osteosarcoma pulmonary metastases. Expert. Rev. Anticancer. Ther..

[56] Kager L., Potschger U., Bielack S. (2010). Review of mifamurtide in the treatment of patients with osteosarcoma. Ther. Clin. Risk. Manag..

[57] Meyers P.A., Schwartz C.L., Krailo M.D., Healey J.H., Bernstein M.L., Betcher D., Ferguson W.S., Gebhardt M.C., Goorin A.M., Harris M. (2008). Osteosarcoma: The addition of muramyl tripeptide to chemotherapy improves overall survival--a report from the Children’s Oncology Group. J. Clin. Oncol..

[58] Chou A.J., Kleinerman E.S., Krailo M.D., Chen Z., Betcher D.L., Healey J.H., Conrad E.U., Nieder M.L., Weiner M.A., Wells R.J. (2009). Addition of muramyl tripeptide to chemotherapy for patients with newly diagnosed metastatic osteosarcoma: A report from the Children’s Oncology Group. Cancer.

[59] Gabrilovich D.I., Nagaraj S. (2009). Myeloid-derived suppressor cells as regulators of the immune system. Nat. Rev. Immunol..

[60] Santilli G., Piotrowska I., Cantilena S., Chayka O., D’Alicarnasso M., Morgenstern D.A., Himoudi N., Pearson K., Anderson J., Thrasher A.J. (2013). Polyphenon [corrected] E enhances the antitumor immune response in neuroblastoma by inactivating myeloid suppressor cells. Clin Cancer Res..

[61] Liao W., Lin J.X., Leonard W.J. (2013). Interleukin-2 at the crossroads of effector responses, tolerance, and immunotherapy. Immunity.

[62] Konjevic G., Mirjacic Martinovic K., Vuletic A., Babovic N. (2010). In-vitro IL-2 or IFN-alpha-induced NKG2D and CD161 NK cell receptor expression indicates novel aspects of NK cell activation in metastatic melanoma patients. Melanoma Res..

[63] Roper M., Smith M.A., Sondel P.M., Gillespie A., Reaman G.H., Hammond G.D., Levitt D., Rosolen A., Colamonici O.R., Neckers L.M. (1992). A phase I study of interleukin-2 in children with cancer. Am. J. Pediatr. Hematol. Oncol..

[64] Bauer M., Reaman G.H., Hank J.A., Cairo M.S., Anderson P., Blazar B.R., Frierdich S., Sondel P.M. (1995). A phase II trial of human recombinant interleukin-2 administered as a 4-day continuous infusion for children with refractory neuroblastoma, non-Hodgkin’s lymphoma, sarcoma, renal cell carcinoma, and malignant melanoma. A childrens cancer group study. Cancer.

[65] Kalwak K., Ussowicz M., Gorczynska E., Turkiewicz D., Toporski J., Dobaczewski G., Latos-Grazynska E., Ryczan R., Noworolska-Sauren D., Chybicka A. (2003). Immunologic effects of intermediate-dose IL-2 i.v. after autologous hematopoietic cell transplantation in pediatric solid tumors. J. Interferon Cytokine Res..

[66] Hakansson A., Gustafsson B., Krysander L., Hakansson L. (1996). Tumour-infiltrating lymphocytes in metastatic malignant melanoma and response to interferon alpha treatment. Br. J. Cancer.

[67] Navid F., Furman W.L., Fleming M., Rao B.N., Kovach S., Billups C.A., Cain A.M., Amonette R., Jenkins J.J., Pappo A.S. (2005). The feasibility of adjuvant interferon alpha-2b in children with high-risk melanoma. Cancer.

[68] Warren K., Bent R., Wolters P.L., Prager A., Hanson R., Packer R., Shih J., Camphausen K. (2012). A phase 2 study of pegylated interferon alpha-2b (PEG-Intron((R))) in children with diffuse intrinsic pontine glioma. Cancer.

[69] Jakacki R.I., Dombi E., Steinberg S.M., Goldman S., Kieran M.W., Ullrich N.J., Pollack I.F., Goodwin A., Manley P.E., Fangusaro J. (2017). Phase II trial of pegylated interferon alfa-2b in young patients with neurofibromatosis type 1 and unresectable plexiform neurofibromas. Neuro Oncol..

[70] Kohli S.S., Kohli V.S. (2011). Role of RANKL-RANK/osteoprotegerin molecular complex in bone remodeling and its immunopathologic implications. Indian J. Endocrinol Metab..

[71] Roodman G.D. (2004). Mechanisms of bone metastasis. N. Engl. J. Med..

[72] Fizazi K., Lipton A., Mariette X., Body J.J., Rahim Y., Gralow J.R., Gao G., Wu L., Sohn W., Jun S. (2009). Randomized phase II trial of denosumab in patients with bone metastases from prostate cancer, breast cancer, or other neoplasms after intravenous bisphosphonates. J. Clin. Oncol..

[73] Lee J.A., Jung J.S., Kim D.H., Lim J.S., Kim M.S., Kong C.B., Song W.S., Cho W.H., Jeon D.G., Lee S.Y. (2011). RANKL expression is related to treatment outcome of patients with localized, high-grade osteosarcoma. Pediatr. Blood Cancer.

[74] Nakano K., Abe S., Sohmura Y. (1986). Recombinant human tumor necrosis factor--I. Cytotoxic activity in vitro. Int. J. Immunopharmacol..

[75] Lejeune F.J., Lienard D., Matter M., Ruegg C. (2006). Efficiency of recombinant human TNF in human cancer therapy. Cancer Immunol..

[76] Seibel N.L., Dinndorf P.A., Bauer M., Sondel P.M., Hammond G.D., Reaman G.H. (1994). Phase I study of tumor necrosis factor-alpha and actinomycin D in pediatric patients with cancer: A Children’s Cancer Group study. J. Immunother. Emphasis Tumor Immunol..

[77] Meany H.J., Seibel N.L., Sun J., Finklestein J.Z., Sato J., Kelleher J., Sondel P., Reaman G. (2008). Phase 2 trial of recombinant tumor necrosis factor-alpha in combination with dactinomycin in children with recurrent Wilms tumor. J. Immunother..

[78] Daniels R.A., Turley H., Kimberley F.C., Liu X.S., Mongkolsapaya J., Ch’En P., Xu X.N., Jin B.Q., Pezzella F., Screaton G.R. (2005). Expression of TRAIL and TRAIL receptors in normal and malignant tissues. Cell Res..

[79] Picarda G., Lamoureux F., Geffroy L., Delepine P., Montier T., Laud K., Tirode F., Delattre O., Heymann D., Redini F. (2010). Preclinical evidence that use of TRAIL in Ewing’s sarcoma and osteosarcoma therapy inhibits tumor growth, prevents osteolysis, and increases animal survival. Clin. Cancer Res..

[80] Petak I., Douglas L., Tillman D.M., Vernes R., Houghton J.A. (2000). Pediatric rhabdomyosarcoma cell lines are resistant to Fas-induced apoptosis and highly sensitive to TRAIL-induced apoptosis. Clin. Cancer Res..

[81] Merchant M.S., Geller J.I., Baird K., Chou A.J., Galli S., Charles A., Amaoko M., Rhee E.H., Price A., Wexler L.H. (2012). Phase I trial and pharmacokinetic study of lexatumumab in pediatric patients with solid tumors. J. Clin. Oncol..

[82] Baek J.H., Sohn S.K., Kim D.H., Kim J.G., Yang D.H., Kim Y.K., Lee J.J., Kim H.J. (2007). Pilot remission induction therapy with idarubicin, plus an intensified dose of ara-C and priming with granulocyte colony-stimulating factor for acute myeloid leukemia. Acta Haematol..

[83] Anderson P.M., Markovic S.N., Sloan J.A., Clawson M.L., Wylam M., Arndt C.A., Smithson W.A., Burch P., Gornet M., Rahman E. (1999). Aerosol granulocyte macrophage-colony stimulating factor: A low toxicity, lung-specific biological therapy in patients with lung metastases. Clin. Cancer Res..

[84] Merchant M.S., Yang X., Melchionda F., Romero M., Klein R., Thiele C.J., Tsokos M., Kontny H.U., Mackall C.L. (2004). Interferon gamma enhances the effectiveness of tumor necrosis factor-related apoptosis-inducing ligand receptor agonists in a xenograft model of Ewing’s sarcoma. Cancer Res..

[85] Cartellieri M., Bachmann M., Feldmann A., Bippes C., Stamova S., Wehner R., Temme A., Schmitz M. (2010). Chimeric antigen receptor-engineered T cells for immunotherapy of cancer. J. Biomed. Biotechnol..

[86] Hombach A., Wieczarkowiecz A., Marquardt T., Heuser C., Usai L., Pohl C., Seliger B., Abken H. (2001). Tumor-specific T cell activation by recombinant immunoreceptors: CD3 zeta signaling and CD28 costimulation are simultaneously required for efficient IL-2 secretion and can be integrated into one combined CD28/CD3 zeta signaling receptor molecule. J. Immunol..

[87] Pule M.A., Straathof K.C., Dotti G., Heslop H.E., Rooney C.M., Brenner M.K. (2005). A chimeric T cell antigen receptor that augments cytokine release and supports clonal expansion of primary human T cells. Mol. Ther..

[88] Zhong X.S., Matsushita M., Plotkin J., Riviere I., Sadelain M. (2010). Chimeric antigen receptors combining 4-1BB and CD28 signaling domains augment PI3kinase/AKT/Bcl-XL activation and CD8+ T cell-mediated tumor eradication. Mol. Ther..

[89] Huang M.A., Krishnadas D.K., Lucas K.G. (2015). Cellular and Antibody Based Approaches for Pediatric Cancer Immunotherapy. J. Immunol. Res..

[90] Louis C.U., Savoldo B., Dotti G., Pule M., Yvon E., Myers G.D., Rossig C., Russell H.V., Diouf O., Liu E. (2011). Antitumor activity and long-term fate of chimeric antigen receptor-positive T cells in patients with neuroblastoma. Blood.

[91] Pule M.A., Savoldo B., Myers G.D., Rossig C., Russell H.V., Dotti G., Huls M.H., Liu E., Gee A.P., Mei Z. (2008). Virus-specific T cells engineered to coexpress tumor-specific receptors: Persistence and antitumor activity in individuals with neuroblastoma. Nat. Med..

[92] Heczey A., Louis C.U. (2013). Advances in chimeric antigen receptor immunotherapy for neuroblastoma. Discov. Med..

[93] Walker A.J., Majzner R.G., Zhang L., Wanhainen K., Long A.H., Nguyen S.M., Lopomo P., Vigny M., Fry T.J., Orentas R.J. (2017). Tumor Antigen and Receptor Densities Regulate Efficacy of a Chimeric Antigen Receptor Targeting Anaplastic Lymphoma Kinase. Mol. Ther..

[94] Orentas R.J., Lee D.W., Mackall C. (2012). Immunotherapy targets in pediatric cancer. Front. Oncol..

[95] Ragab S.M., Samaka R.M., Shams T.M. (2010). HER2/neu expression: A predictor for differentiation and survival in children with Wilms tumor. Pathol. Oncol. Res..

[96] Ahmed N., Salsman V.S., Yvon E., Louis C.U., Perlaky L., Wels W.S., Dishop M.K., Kleinerman E.E., Pule M., Rooney C.M. (2009). Immunotherapy for osteosarcoma: Genetic modification of T cells overcomes low levels of tumor antigen expression. Mol. Ther..

[97] Hegde M., Moll A.J., Byrd T.T., Louis C.U., Ahmed N. (2015). Cellular immunotherapy for pediatric solid tumors. Cytotherapy.

[98] Rainusso N., Brawley V.S., Ghazi A., Hicks M.J., Gottschalk S., Rosen J.M., Ahmed N. (2012). Immunotherapy targeting HER2 with genetically modified T cells eliminates tumor-initiating cells in osteosarcoma. Cancer Gene Ther..

[99] Ahmed N., Brawley V.S., Hegde M., Robertson C., Ghazi A., Gerken C., Liu E., Dakhova O., Ashoori A., Corder A. (2015). Human Epidermal Growth Factor Receptor 2 (HER2) -Specific Chimeric Antigen Receptor-Modified T Cells for the Immunotherapy of HER2-Positive Sarcoma. J. Clin. Oncol..

[100] Kawakami M., Kawakami K., Takahashi S., Abe M., Puri R.K. (2004). Analysis of interleukin-13 receptor alpha2 expression in human pediatric brain tumors. Cancer.

[101] Okada H., Low K.L., Kohanbash G., McDonald H.A., Hamilton R.L., Pollack I.F. (2008). Expression of glioma-associated antigens in pediatric brain stem and non-brain stem gliomas. J. Neurooncol..

[102] Brown C.E., Starr R., Aguilar B., Shami A.F., Martinez C., D’Apuzzo M., Barish M.E., Forman S.J., Jensen M.C. (2012). Stem-like tumor-initiating cells isolated from IL13Ralpha2 expressing gliomas are targeted and killed by IL13-zetakine-redirected T Cells. Clin. Cancer Res..

[103] Kahlon K.S., Brown C., Cooper L.J., Raubitschek A., Forman S.J., Jensen M.C. (2004). Specific recognition and killing of glioblastoma multiforme by interleukin 13-zetakine redirected cytolytic T cells. Cancer Res..

[104] Brudno J.N., Kochenderfer J.N. (2019). Recent advances in CAR T-cell toxicity: Mechanisms, manifestations and management. Blood Rev..

[105] Teachey D.T., Lacey S.F., Shaw P.A., Melenhorst J.J., Maude S.L., Frey N., Pequignot E., Gonzalez V.E., Chen F., Finklestein J. (2016). Identification of Predictive Biomarkers for Cytokine Release Syndrome after Chimeric Antigen Receptor T-cell Therapy for Acute Lymphoblastic Leukemia. Cancer Discov..

[106] Maude S.L., Barrett D., Teachey D.T., Grupp S.A. (2014). Managing cytokine release syndrome associated with novel T cell-engaging therapies. Cancer J..

[107] Straathof K.C., Pule M.A., Yotnda P., Dotti G., Vanin E.F., Brenner M.K., Heslop H.E., Spencer D.M., Rooney C.M. (2005). An inducible caspase 9 safety switch for T-cell therapy. Blood.

[108] Di Stasi A., Tey S.K., Dotti G., Fujita Y., Kennedy-Nasser A., Martinez C., Straathof K., Liu E., Durett A.G., Grilley B. (2011). Inducible apoptosis as a safety switch for adoptive cell therapy. N. Engl. J. Med..

[109] Boyton R.J., Altmann D.M. (2007). Natural killer cells, killer immunoglobulin-like receptors and human leucocyte antigen class I in disease. Clin. Exp. Immunol..

[110] Leung W., Iyengar R., Turner V., Lang P., Bader P., Conn P., Niethammer D., Handgretinger R. (2004). Determinants of antileukemia effects of allogeneic NK cells. J. Immunol..

[111] Koscielniak E., Gross-Wieltsch U., Treuner J., Winkler P., Klingebiel T., Lang P., Bader P., Niethammer D., Handgretinger R. (2005). Graft-versus-Ewing sarcoma effect and long-term remission induced by haploidentical stem-cell transplantation in a patient with relapse of metastatic disease. J. Clin. Oncol..

[112] Lang P., Pfeiffer M., Muller I., Schumm M., Ebinger M., Koscielniak E., Feuchtinger T., Foll J., Martin D., Handgretinger R. (2006). Haploidentical stem cell transplantation in patients with pediatric solid tumors: Preliminary results of a pilot study and analysis of graft versus tumor effects. Klin. Padiatr..

[113] Kasow K.A., Handgretinger R., Krasin M.J., Pappo A.S., Leung W. (2003). Possible allogeneic graft-versus-tumor effect in childhood melanoma. J. Pediatr. Hematol. Oncol..

[114] Inaba H., Handgretinger R., Furman W., Hale G., Leung W. (2006). Allogeneic graft-versus-hepatoblastoma effect. Pediatr. Blood Cancer.

[115] Perez-Martinez A., Leung W., Munoz E., Iyengar R., Ramirez M., Vicario J.L., Lassaletta A., Sevilla J., Gonzalez-Vicent M., Madero L. (2009). KIR-HLA receptor-ligand mismatch associated with a graft-versus-tumor effect in haploidentical stem cell transplantation for pediatric metastatic solid tumors. Pediatr. Blood Cancer.

[116] Ibrahim E.C., Guerra N., Lacombe M.J., Angevin E., Chouaib S., Carosella E.D., Caignard A., Paul P. (2001). Tumor-specific up-regulation of the nonclassical class I HLA-G antigen expression in renal carcinoma. Cancer Res..

[117] Wan R., Wang Z.W., Li H., Peng X.D., Liu G.Y., Ou J.M., Cheng A.Q. (2017). Human Leukocyte Antigen-G Inhibits the Anti-Tumor Effect of Natural Killer Cells via Immunoglobulin-Like Transcript 2 in Gastric Cancer. Cell. Physiol. Biochem..

[118] Kailayangiri S., Altvater B., Spurny C., Jamitzky S., Schelhaas S., Jacobs A.H., Wiek C., Roellecke K., Hanenberg H., Hartmann W. (2017). Targeting Ewing sarcoma with activated and GD2-specific chimeric antigen receptor-engineered human NK cells induces upregulation of immune-inhibitory HLA-G. Oncoimmunology.

[119] Maki G., Hayes G.M., Naji A., Tyler T., Carosella E.D., Rouas-Freiss N. (2008). Gregory SANK resistance of tumor cells from multiple myeloma chronic lymphocytic leukemia patients: Implication of, H.L.A.-G. Leukemia.

[120] Hutzen B., Ghonime M., Lee J., Mardis E.R., Wang R., Lee D.A., Cairo M.S., Roberts R.D., Cripe T.P., Cassady K.A. (2019). Immunotherapeutic Challenges for Pediatric Cancers. Mol. Ther. Oncolytics.

[121] Elster J.D., Krishnadas D.K., Lucas K.G. (2016). Dendritic cell vaccines: A review of recent developments and their potential pediatric application. Hum. Vaccin. Immunother..

[122] Jarnjak-Jankovic S., Hammerstad H., Saeboe-Larssen S., Kvalheim G., Gaudernack G. (2007). A full scale comparative study of methods for generation of functional Dendritic cells for use as cancer vaccines. BMC Cancer.

[123] Fields R.C., Shimizu K., Mule J.J. (1998). Murine dendritic cells pulsed with whole tumor lysates mediate potent antitumor immune responses in vitro and in vivo. Proc. Natl. Acad. Sci. USA.

[124] Meadors J.L., Cui Y., Chen Q.R., Song Y.K., Khan J., Merlino G., Tsokos M., Orentas R.J., Mackall C.L. (2011). Murine rhabdomyosarcoma is immunogenic and responsive to T-cell-based immunotherapy. Pediatr. Blood Cancer.

[125] Geiger J.D., Hutchinson R.J., Hohenkirk L.F., McKenna E.A., Yanik G.A., Levine J.E., Chang A.E., Braun T.M., Mule J.J. (2001). Vaccination of pediatric solid tumor patients with tumor lysate-pulsed dendritic cells can expand specific T cells and mediate tumor regression. Cancer Res..

[126] Krishnadas D.K., Shapiro T., Lucas K. (2013). Complete remission following decitabine/dendritic cell vaccine for relapsed neuroblastoma. Pediatrics.

[127] Krishnadas D.K., Shusterman S., Bai F., Diller L., Sullivan J.E., Cheerva A.C., George R.E., Lucas K.G. (2015). A phase I trial combining decitabine/dendritic cell vaccine targeting MAGE-A1, MAGE-A3 and NY-ESO-1 for children with relapsed or therapy-refractory neuroblastoma and sarcoma. Cancer Immunol. Immunother..

